# COVID-19 Infection Segmentation and Severity Assessment Using a Self-Supervised Learning Approach

**DOI:** 10.3390/diagnostics12081805

**Published:** 2022-07-26

**Authors:** Yao Song, Jun Liu, Xinghua Liu, Jinshan Tang

**Affiliations:** 1 School of Computer Science and Technology, Wuhan University of Science and Technology, Wuhan 430065, China; i_songyao@163.com; 2 Hubei Province Key Laboratory of Intelligent Information Processing and Real-Time Industrial System, Wuhan 430065, China; 3 Wuhan First Hospital, Wuhan 430030, China; huxinghua0711@126.com; 4 Department of Health Administration and Policy, College of Health and Human Services, George Mason University, Fairfax, VA 22030, USA

**Keywords:** self-supervised learning, COVID-19, lesion segmentation

## Abstract

Background: Automated segmentation of COVID-19 infection lesions and the assessment of the severity of the infections are critical in COVID-19 diagnosis and treatment. Based on a large amount of annotated data, deep learning approaches have been widely used in COVID-19 medical image analysis. However, the number of medical image samples is generally huge, and it is challenging to obtain enough annotated medical images for training a deep CNN model. Methods: To address these challenges, we propose a novel self-supervised deep learning method for automated segmentation of COVID-19 infection lesions and assessing the severity of infection, which can reduce the dependence on the annotation of the training samples. In the proposed method, first, many unlabeled data are used to pre-train an encoder-decoder model to learn rotation-dependent and rotation-invariant features. Then, a small amount of labeled data is used to fine-tune the pre-trained encoder-decoder for COVID-19 severity classification and lesion segmentation. Results: The proposed methods were tested on two public COVID-19 CT datasets and one self-built dataset. Accuracy, precision, recall, and F1-score were used to measure classification performance and Dice coefficient was used to measure segmentation performance. For COVID-19 severity classification, the proposed method outperformed other unsupervised feature learning methods by about 7.16% in accuracy. For segmentation, when the amount of labeled data was 100%, the Dice value of the proposed method was 5.58% higher than that of U-Net.; in 70% of the cases, our method was 8.02% higher than U-Net; in 30% of the cases, our method was 11.88% higher than U-Net; and in 10% of the cases, our method was 16.88% higher than U-Net. Conclusions: The proposed method provides better classification and segmentation performance under limited labeled data than other methods.

## 1. Introduction

Currently, the novel coronavirus disease 2019 (COVID-19) is spreading rapidly around the world, seriously affecting people’s daily life. The gold standard for detecting COVID-19 is reverse transcription-polymerase chain reaction (RT-PCR) [1], which uses the combination of RNA reverse transcription and polymerase chain reaction to detect viral RNA fragments. However, RT-PCR tests consume time to obtain the results and have relatively low sensitivity, which does not meet the appeal of detecting positive cases as quickly as possible to separate the persons with positive tests. Another solution for rapid screening is to use medical imaging, such as chest X-ray images or computed tomography (CT) scanners [2]. In addition, some methods have also been proposed for the treatment of COVID-19 [3,4]. In medical image analysis, many image-based artificial intelligence approaches have been developed to help combat the disease, including automatic diagnosis, segmentation, and prognosis. However, most previous image-based studies focused on identifying COVID-19 patients. Moreover, approximately 80% of patients with COVID-19 have only mild to moderate symptoms, and only 20% have severe symptoms [5,6]. Therefore, there is no doubt that automatic assessment of COVID-19 severity is of great significance for clinical diagnosis. At the same time, the segmentation of new coronary pneumonia lesions in lung CT images can also assist doctors in diagnosing the disease.

In recent years, deep learning has achieved great success in medical image analysis. The convolutional neural network’s (CNN) success is mainly due to its ability to extract highly representative features. However, it relies heavily on many high-quality manual annotations. There are issues when manual annotations are used: expensive acquisition costs and patient data privacy. To alleviate the challenge of complex medical image annotation, several solutions were provided in the past:

1. Transfer learning [7] was used to fine-tune the pre-trained model for the target task. However, transfer learning can be impractical and inefficient because the predefined model’s architecture is not as accurate as transfer learning from medical images because of the differences in image features.
2. Semi-supervised learning [8] was proposed to exploit an unlimited amount of unlabeled data to improve performance. However, it usually assumes that labeled data is sufficient to cover the data distribution, with smoothness assumptions, low-density separation assumptions, and popular assumptions, such as adversarial learning [9] and noisy labels [10].
3. Active learning [11] was developed to select the most representative samples that are annotated without unannotated data but only focus on saving manual labor.
4. Self-Supervised Learning [12], also known as unsupervised visual representation learning, can be achieved by providing a strategy to pre-train the neural network with unlabeled data, draw the prior knowledge distribution of the image itself, and then fine-tune the downstream tasks with limited annotation to transfer the knowledge learned during the pre-training process to the downstream tasks and improve the downstream tasks.

In this paper, we developed a new SSL approach. Our new approach first pre-trains a convolutional neural network on a pretext task with a large unannotated dataset. It then uses a small set of annotated data to fine-tune the pre-trained network for a specific target task. The pre-task uses a neural network to deeply mine useful information from the unlabeled raw data to improve the performance of subsequent target tasks when the training data is limited.

Various pretext tasks have been proposed, including grayscale image colorization prediction [13], puzzle prediction [14], object motion and depth estimation [15], rotation prediction [16], etc. Self-supervision in the image domain can be divided into generative self-supervised learning and discriminative self-supervised learning. Variational auto-encoder (VAE) [17] and generative adversarial network (GAN) [18] are generative self-supervised learning, while contrastive learning (CL) is a typical discriminative self-supervised learning. Contrastive learning defines a contrastive prediction task, which namely attempts to maximize the similarity between the features in different augmented views of the same image through a contrastive loss while maximizing the distance between the features in different images. Studies have shown that representations learned through contrastive learning gradually approach learning through strong supervision to the characterization, even when only limited data or small-scale datasets are available. Various types of self-supervised methods have been developed for a variety of medical applications, such as object recognition for spine MRI [19], cardiac MR image segmentation [20], lung lobe segmentation and nodule detection [21], intracerebral hemorrhage classification, brain tumors segmentation [22,23], etc.

**Figure 1:**
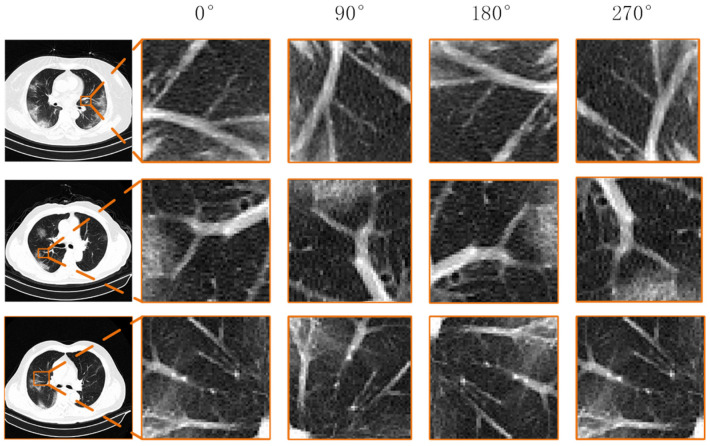
Example of blood vessel orientation and rotation in CT images of COVID-19 patients.

**Figure 2:**
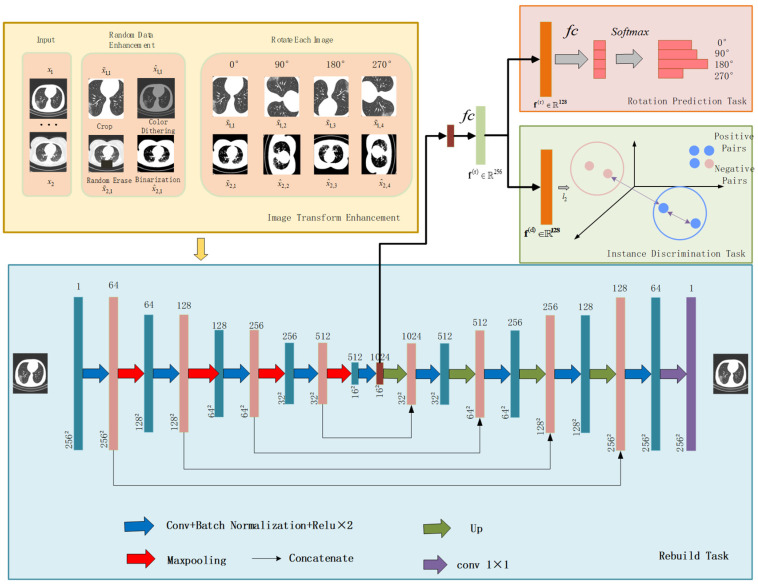
The framework of the proposed method.

Inspired by the rotation-dependent and rotation-invariant features [24,25,26,27], we formulate a rotation prediction task and instance discrimination task to learn rotation-dependent features through rotation transformations and identify instances without considering image rotations. We observed that the blood vessels in the pneumonia image are often interlaced with ground glass or consolidation, which is very sensitive to the direction. The blood vessels interspersed by the lesion have a specific direction, as shown in Figure 1, and the CT image was rotated by 90°, 180°, and 270°. Afterwards, the apparent orientation of these structures will be changed. Therefore, learning the predicted rotation helps to learn the deep features of pneumonia images, which is beneficial for self-supervised feature learning while improving the accuracy of downstream diagnostic tasks. However, CNNs with pooling layers are less sensitive to some spatial transformations, such as positional changes, for segmentation tasks. Furthermore, such spatially invariant features are advantageous for classification problems but a disadvantage for segmentation problems. So, in addition to learning salient features, we also propose a multi-view instance discrimination task to learn rotation-invariant features. As shown in Figure 2, the multi-view instance discrimination task aims to learn a feature representation such as the representation of the transformed version of the input image but different from other images, where transformations include random cropping, color dithering, random erasing, and binarization. By formulating collaborative learning tasks, namely rotation prediction and multi-view instance discrimination, we propose the network to discover the discriminative structure of pneumonia CT images and explore for diagnosis and lesion segmentation of COVID-19 severity. Finally, after appending a decoder with an encoder, we pre-train the encoder-decoder architecture to obtain proper initializations for downstream segmentation tasks.

In summary, our main contributions are three-fold.

1. We propose a novel SSL framework that provides an encoder for classification and an encoder-decoder for segmentation for severity classification and lesion segmentation from COVID-19 CT images.
2. We formulate a collaborative learning task that splits features into learning rotation-dependent and invariant representations, which can learn rotation-discriminative features from pneumonia images and rotation-independent features. When comparing prediction tasks, we investigate different data augmentation operations in the definition. The results show that random cropping, color dithering, random erasing, and binarization combined are helpful for the pretext task of CT images to learn discriminative feature representations.
3. Experiments are conducted on an aggregated dataset consisting of three COVID-19 CT image datasets. The results show that our self-supervised method achieved better results than other state-of-the-art methods. Our method has demonstrated superior performance in COVID-19 severity diagnosis and lesion segmentation. Our method can reduce the performance gaps caused by the number of annotated datasets (10% vs. 100% annotations) with limited annotated datasets.

## 2. Related Works

### 2.1. Deep Learning for COIVD-19 Diagnosis Based on Classification and Segmentation Tasks

Deep learning-based methods have been widely used in the automatic diagnosis and lesion segmentation of COVID-19 [28,29,30]. For example, Qian et al. [31] proposed a multi-task multi-slice deep learning system (M3 Lung-Sys) for multi-type lung pneumonia screening from CT images. The system consists of only two 2D CNN networks. The former is designed to find feature representations from rich CT slices, and the latter can recover temporal information through feature refinement and aggregation between different slices. In addition to linking COVID-19 with health, H1N1 and CAP cases are distinguished, and the system can also locate the area of related lesions. Zhang et al. [32] proposed a detection and diagnosis model based on EfficientNet, using the EfficientNet-B0 network to pre-train ImageNet to extract features. An anomaly detection module and a confidence score prediction module was proposed to classify COVID-19 and non-COVID-19 patients. Liu et al. [33] proposed a new weakly supervised segmentation method for COVID-19 infection in CT slices, which only requires scribble supervision and is enhanced by uncertainty-aware self-integration and transformation consistency techniques. Wu et al. [34] proposed a sequential region generation network (SRGNet) to detect and segment the lesion areas of COVID-19 jointly. SRGNet can use the supervised segmentation information and then outputs multi-scale segmentation predictions and generates high-quality lesion area proposals on the predicted segmentation map. At the same time, the detection results, in turn, refine the segmentation map through the post-processing process, which significantly improves the segmentation accuracy.

In the application of COVID-19 classification and segmentation, a few works were devoted to the severity assessment of COVID-19. For example, He et al. [5] proposed a synergistic learning framework for automated severity assessment of COVID-19 in 3D CT images by jointly performing lung lobe segmentation and multi-instance classification. Goncharov et al. [35] proposed a new convolutional neural network model. ResNet50 [36] was trained as a classification network in their model, and the distances between the voxels were finally used to segment the lesion area based on U-Net [37]. The proportion of the lesion area in the lung area corresponds to a different severity degree. However, these works are based on supervised or weakly supervised learning, which adopts a large amount of labeled data for training, requiring a lot of time. Unlike previous work, this paper proposes a self-supervised method, which makes full use of a large amount of unlabeled data and reduces the labeling work of doctors.

### 2.2. Self-Supervised Learning

Self-supervised learning is a widely studied area [38,39], and Doersch et al. [40] proposed a framework to learn visual features by predicting the relative positions of two patches from the same image. Another representative method for relative position prediction is the jigsaw puzzle proposed by Noroozi [14]. This work requires a deep learning network to rearrange the positions of nine patches cropped from the same image. In addition, colorization [41] can also be formulated as a pretext task for pre-trained neural networks. Recently, models with SSL ideas began to be gradually applied in the field of medical images, and pretext tasks used in medical images include Rubik’s Cube and Rubik’s Cube + Recovery, anatomical location prediction, and reconstructing a part of an image. For example, Zhuang et al. [22] proposed a self-supervised learning framework for 3D medical images. It proposes a new pretext task-Rubik’s Cube Restoration, i.e., cube rearrangement, and cube rotation, to pre-train a 3D neural network that learns translation and rotation invariant features from raw data for subsequent intracerebral hemorrhage classification and brain tumor segmentation task. Zhu et al. [23] further improved this method. They proposed a new pretext task, adding a masking recognition pretext task based on the two pretext tasks of Zhuang [22], which increased the difficulty of recovering the Rubik’s Cube. Deep learning networks are encouraged to utilize more spatial information and produce more robust feature representations. Bai et al. [20] formulated an anatomical location prediction pretext task to learn self-supervised features for cardiac MR image segmentation. Liang et al. [42] proposed a novel self-supervised learning strategy based on context recovery, which allows the model to learn a priori visual features by reconstructing the original image, which is then applied to many medical image tasks. Recently, contrastive learning methods based on instance discrimination tasks have achieved state-of-the-art performance in SSL. The main idea of contrastive learning methods is to make representations of different views of the same representations (“negative pairs”) of the views of the image that are separated. For example, Dosovitskiy et al. [43] proposed to use SoftMax embeddings with classifier weights to compute feature similarity. However, it prevents the explicit comparison of features, which leads to limited efficiency and discriminability. Wu et al. [44] developed a memory bank to memorize the features of each instance. Ye [45] computes positive set attributes based on “real” instance features rather than classifier weights or the memory bank. There are also some self-supervisions in COVID-19 learning work. Chen et al. [46] proposed a self-supervised learning method. They used contrastive learning to train an encoder that could capture expressive feature representations on a large publicly available lung dataset. A prototype network is used for the classification of the task. We propose a new collaborative method to learn complementary information from different pretext tasks, i.e., rotation-related features, and rotation-invariant features, to better use unlabeled data and improve model performance in generalization ability on downstream tasks.

## 3. Materials and Methods

The overall architecture of the proposed self-supervised method for assessing the severity of COVID-19 is shown in Figure 2. The proposed self-supervised method comprises four modules: image transform enhancement module, rebuild task module, instance discrimination task module, and rotation prediction task module.

This paper randomly selects *m* slices from the training dataset $S={\left\{x_{i}\right\}}_{i=1}^{N}$. For each image slice $x_{i}$, two new images, ${\hat{x}}_{i}$ and ${\tilde{x}}_{i}$, are generated from it by applying two of the four image enhancement techniques. The enhanced image of slides $x_{1}$ and $x_{2}$ are shown in Figure 2. Then, these images are rotated at 0°, 90°, 180°, and 270°to further generate 8 rotated images (denoted by $x_{i}$). After that, each rotated image is assigned a corresponding rotation label 0, 1, 2, and 3. On this basis, a feature embedding network F(⋅;θ) is proposed, which maps the input $x_{i}$ to a high-dimensional feature vector $f_{i}$ and then is decoupled into $f_{i}^{\left(d\right)}$ and $f_{i}^{\left(r\right)}$. These two decoupled features are co-optimized by the multi-view instance discrimination and rotation prediction tasks. Finally, the features learned from the rotation prediction task are utilized for COVID-19 severity classification. The following subsection will introduce the rotation prediction task, multi-view instance discrimination task, reconstruction task, and encoder-decoder details in detail.

### 3.1. Rotation Prediction Task

To discover the salient features of COVID-19 CT images, we use the rotation prediction task module to learn rotation-related features. The input $x_{i}$ is the input to the encoder, and we represent the output of the last convolutional layer as a feature $f_{i}$. The input $x_{i}$ is rotated to get $x_{i,y}$; the output $f_{i}$ feature should be $f_{i,y}$, y∈{0,1,2,3}. To simplify the description, here, $f_{i}$ is used instead of $f_{i,y}$. Then, to reduce the feature dimension and obtain a high-level representation, $f_{i}$ is followed by a module, which is FC, BN, and ReLU. Then, $f_{i}$ is decoupled into $f_{i}^{\left(d\right)}$ and $f_{i}^{\left(r\right)}$ along with the channel layer, respectively. Finally, a fully connected layer is denoted as $F_{c}\left(\cdot ;\theta_{c}\right)$, inputs $f_{i}^{\left(r\right)}$, and after a Softmax operation, outputs four probability values corresponding to 0, 1, 2, and 3. The rotation prediction loss is expressed as:

(1) $${ℒ}_{r}=\frac{1}{4N}\sum_{i=1}^{N}\sum_{y=0}^{3}l\left(F_{c}\left(f_{i,y}^{\left(r\right)};\theta_{c}\right),y\right).$$

where is the cross-entropy loss for the classification task and is the rotation label.

### 3.2. Multi-View Instance Discrimination Task

To learn rotation and transformation invariant representations for COVID-19 lesion segmentation, we proposed a multi-view instance discrimination task. As shown in Figure 2, ${\tilde{x}}_{i,y}$, ${\hat{x}}_{i,y}$ represent different data-augmented views of $x_{i}$. The key assumption of the multi-view instance discrimination task is that good features are shared among the various views of the same image. Therefore, the key is that different data-augmented views (front-facing) of a single image should remain invariant in the embedding space, while different data-augmented views from different patient’s images (negative pair) should be discrepant. After obtaining the decoupling feature $f_{i}^{\left(d\right)}$, this paper first uses $l_{2}$ to normalize to make $\|f_{i}^{\left(d\right)}{\|}_{2}=1$. For simplicity, in this section, this paper uses $f_{i}$ to represent $f_{i}^{\left(d\right)}$, positive pairs are denoted as $\left({\hat{f}}_{i,y},{\tilde{f}}_{i,k}\right)$, y and k are denoted rotation labels, y,k∈{0,1,2,3}, and negative pairs are denoted as $\left({\hat{f}}_{i,y},{\tilde{f}}_{j,k}\right)$, i≠j, as shown in Figure 2. For each image $x_{i}$, the augmented sample should be classified into class i, and other images from ${\hat{x}}_{i,y}$ cannot be classified into class *i*. For X, the probability of being classified as class i is:

(2) $$P(i|{\hat{x}}_{i,y})=\frac{\;\exp\;(\sum_{k=0}^{3}{\tilde{f}}_{i,y}^{T}{\hat{f}}_{i,k}/\tau )}{\sum_{j=1}^{m\sum \sum_{k=0}^{3}{\tilde{f}}_{j,k}^{T}{\hat{f}}_{i,y}}\;exp\;}$$

In the above formula, τ is the temperature parameter, and by default, τ is set to 0.1. ${\tilde{f}}_{i,y}^{T}{\hat{f}}_{i,k}$ represents the cosine similarity between positive pairs, and ${\tilde{f}}_{j,k}^{T}{\hat{f}}_{i,y}$ represents the cosine similarity between negative pairs. Embedding through Softmax in Equation (2) function, the network pushes “negative pairs” from different samples away and pulls “positive pairs” from the same sample closer. The goal is to minimize the contrastive loss, as described below:

(3) $$\begin{array}{c} \begin{array}{c} {ℒ}_{d}=-\underset{i}{\sum }\underset{y}{\sum }logP(i|{\hat{x}}_{i,y})-\\ \underset{i}{\sum }\underset{j\neq i}{\sum }\underset{y}{\sum }log\{(1-P(i|{\tilde{x}}_{j,y}))\} \end{array} \end{array}$$

$P(i|{\hat{x}}_{i,y})$ represents the probability that ${\hat{x}}_{i,y}$ is classified into class i, and $1-P(i|{\tilde{x}}_{j,y})$ represents the probability that ${\tilde{x}}_{j,y}$ is not classified into class i.

### 3.3. Reconstruction Task

To obtain a suitable initialization for the downstream segmentation task, this paper formulates a reconstruction pretext task using the encoder-decoder structure. The loss function is defined as:

(4) $${ℒ}_{\mathrm{rec}\;}=\frac{1}{m}\underset{I\in D}{\sum }\|S(E\left(x_{i}\right))-x_{i}{\|}_{2}$$

E(⋅) is the encoder, S(⋅) is the decoder, and $\|\cdot {\|}_{2}$ is the $L_{2}$ regular.

### 3.4. Loss Function

In summary, we combine the encoder rotation prediction loss, the multi-view discriminative loss, and the encoder-decoder auxiliary reconstruction loss, taking their weighted sum as the final total loss function, and jointly optimize the model. The final loss function is:

(5) $$ℒ=\lambda_{1}{ℒ}_{d}+\lambda_{2}{ℒ}_{r}+\lambda_{3}{ℒ}_{\mathrm{rec}}$$

where $\lambda_{1},\;\lambda_{2}$, $\mathrm{and}\;\lambda_{3}$ are the weights, which represent the importance ratio of each task. In the experiment, we set $\lambda_{1}=1.5,\;\lambda_{2}=1$, $\mathrm{and}\;\lambda_{3}=3$.

### 3.5. Encoder-Decoder Architecture

We take U-Net as the basic encoder-decoder network for self-supervised pre-training. As shown in Figure 2, the encoder is mainly composed of convolutional, activation, and pooling layers. The encoder extracts the effective features of the input image and gradually reduces the spatial dimension of the input data, using max-pooling after the output of the last convolutional layer in the encoder. Then, the features are flattened into a vector and passed through the FC layer, BN, and ReLU, in turn, to reduce the feature dimension to 256.After that, we split and learn the rotation prediction task and the multi-view discrimination task. A layer fully connected to output channel 4 is applied to generate probabilities for each rotation type, while a normalization layer is used to compute the cosine similarity between the features. The decoder mainly consists of convolutional, activation, and up-sampling layers.

The decoder gradually recovers the size and spatial dimension of the image through an up-sampling layer. There is information stitching between the encoder and decoder for fusing low-level and high-level semantic features to help the decoder better recover image details. The detailed network architecture is shown in Table 1.

## 4. Experiments and Results

### 4.1. Datasets

We conducted experiments on two public COVID-19 datasets and a self-built COVID-19 dataset to evaluate the effectiveness of our method.

#### 4.1.1. Self-Built Dataset (3D-COVID)

**Figure 3:**
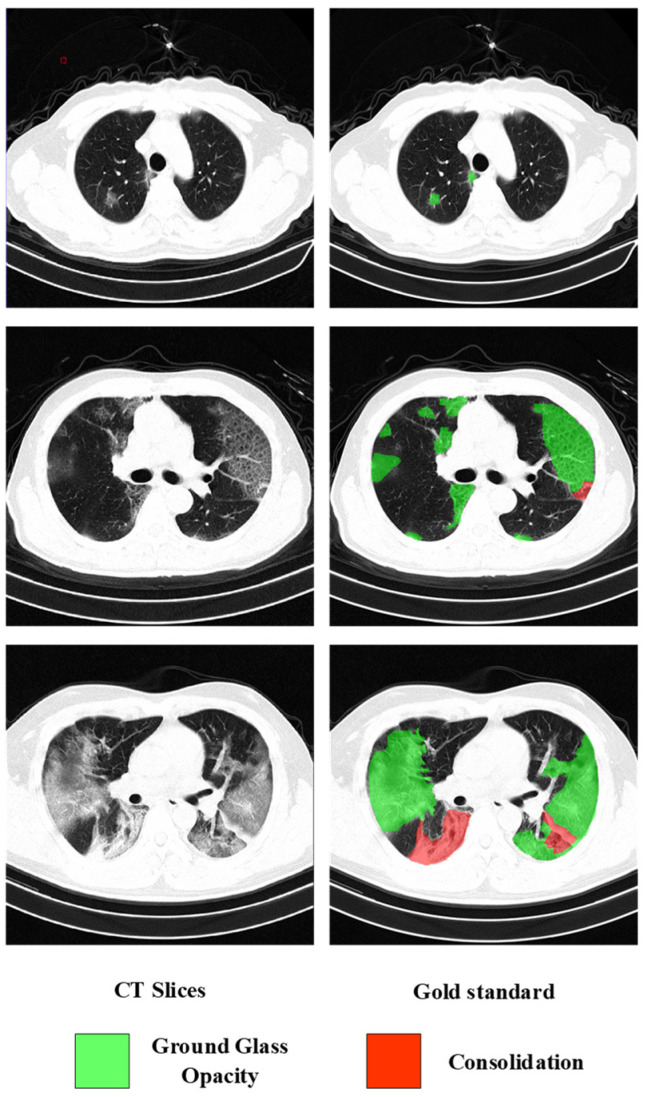
Three examples of CT images of different severity.

The 3D COVID-19 CT dataset contained 2722 3D chest CT slices from 146 patients with confirmed COVID-19 (i.e., positive RT-PCR test). We collected the 3D CT dataset from Wuhan First Hospital. The image size varies from 512×512×233 to 512×512×395, and the spatial resolution is 0.78125×0.78125×1.25 mm. Diagnosis based on these images is a very challenging task. In the work of He et al. [5], the severity of patients was divided into severe and non-severe. In this paper, the severity is defined the same as [47] and is divided into mild, moderate, and severe, as follows: mild is defined as containing less than 3 GGO lesions less than 3 cm in size; moderate is defined as; and severe is defined as the lesion area exceeding 50% of the entire lung field, as shown in Figure 3. Therefore, the task of severity assessment was modeled as a three-category problem. Since the labeling work is very time-consuming, to verify the effectiveness of SSL, 600 slices from the dataset were annotated at the pixel level. A radiologist completed all annotations. In this paper, the image intensity values of all slices were truncated to the HU range [−800,100] to remove irrelevant details. To improve the efficiency of network training, all slices were resized to 256 × 256 after data augmentation.

#### 4.1.2. Lesion Segmentation Dataset (COVID19-Seg)

The second dataset was from Radiopedia (http://medicalsegmentation.com/covid19/, accessed on 1 February 2022). The first version of this dataset contained 100 axial CT images of 40 patients with COVID-19. The size of the original CT images and all ground truth masks was 512 × 512. The second version of the dataset was expanded to 829 images (from nine patients), of which 373 were labeled as COVID-19, and the rest were marked as normal. In this paper, only 373 CT images marked with COVID-19 were used in the second part. The two versions were merged for a total of 473 samples. The dataset provided three image-level lesion labels: ground-glass, consolidation, and pleural effusion, and only ground-glass and consolidation ground-truth segmentation masks were used in this paper. Image-level severity labeling was done by doctors, and 100 images were annotated in this dataset.

#### 4.1.3. Lesion Segmentation Dataset (CC-COVID)

The dataset was a large CT dataset from the Chinese Consortium for Chest CT Image Investigation (CC-CCII), which included a total of 617,775 CT images from 4154 patients. These included 752 NCP patients, 797 common pneumonia patients, and 697 normal control patients. This paper adopted 800 CT slices of size 512 × 512 from 100 COVID-19 patients. After data augmentation, the original slices were resized to 256 × 256 as the input of the network in this paper. We randomly selected 100 CT slices labeled, and the rest were unlabeled data.

### 4.2. Experimental Details

The CT images from the three different datasets were combined into one dataset, denoted as $X_{\mathrm{all}}$, and the labeled datasets are denoted as $X_{l}$. In the pretext task stage, all unlabeled datasets $X_{down}$ were used, and then the labeled images from $X_{down}$ were used to fine-tune the pre-processing and train the network for classification and segmentation tasks. To verify the effect of self-supervised learning, this paper fine-tunes with different numbers of labeled datasets $X_{down}^{s}$, where s∈{10%,30%,70%, 100%}, represents the ratio $\frac{X_{down}^{s}}{X_{down}}$. The training, validation, and testing datasets in the fine-tuning stage were performed according to 7:2:1, respectively. The training, validation, and testing datasets do not contain images from the same patients. The number of sample distributions for the labeled datasets is shown in Table 2. In each training iteration, m images were randomly selected, and two random data augmentations were applied to the selected images to generate 2 m images. Then, each image was rotated by 0°, 90°, 180°, and 270°, thus 8 m images were generated. The final batch size was 8 m. In this experiment, m was set to 16. Data augmentation is a technique widely used in deep learning. As shown in Figure 2, four augmentation operations were used in this paper: random cropping, color dithering, random erasing, and binarization. Random clipping was set to center clipping and adjusted to 256 × 256. Color dithering adjusted the brightness, saturation, contrast, and hue of the image. In our experiment, the range of brightness dithering was set to 0.7, saturation was set to 0.7, contrast was set to 0.4, and hue was set to 0.5. The minimum ratio of the erased area to the input image in random erasing was set to 0.1, and the maximum ratio was set to 0.2. The threshold for binarization was set to 125. The algorithm in this paper was implemented in Python and Pytorch, and the environment was Intel (R) Core (TM) i7 3.40 G CPU, NVIDIA 2080Ti graphics card, 32 G DDR4 RAM. This paper adjusted the image resolution to 256 × 256, and the Adam optimizer was used for network optimization. The learning rate was set to 0.0001, and the weight decay was set to 0.1.

### 4.3. Assessment of Performance

We employed accuracy, precision, recall, and F1-score to measure classification performance and Dice coefficient to measure segmentation performance. The definitions are as follows:

(6) $$\mathrm{Accuracy}=\frac{TP+TN}{TP+TN+FP+FN}$$

(7) $$Precision=\frac{TP}{TP+FP}$$

(8) $$Recall=\frac{TP}{TP+FN}$$

(9) $$F1\;Score=\frac{2\times Precision\times Recall\;}{Precision+Recall\;}$$

(10) $$Dice=\frac{2\left|A\cap B\right|}{\left|A\right|+\left|B\right|}$$

where TP, TN, FP, and FN refer to true positives, true negatives, false positives, and false negatives, respectively; A is the set of segmentation result pixels; and B is the set of actual dataset label pixels.

### 4.4. Classification Results of COVID-19 Severity

The method was compared with some advanced supervised learning methods and self-supervised learning methods. The results are shown in Table 3. For the supervised learning models, this paper was compared with VGG18 [48], ResNet50 [36], and DenseNet121 [49]; we modified the output channels of the last fully connected layer of these models to 3 and trained the models using cross-entropy loss for three-classification with all labeled data. All methods are applied to the same testing database. The paper also compared the proposed algorithm with other self-supervised methods, including Rotation prediction method [50], instance discrimination method [44], Moco V1 [51], and SimCLR [52]. For the rotation prediction method [27], this paper modified the output channel of the last fully connected layer of ResNet18 to 4 and a cross-entropy loss was used to train the network to make predictions for four rotation types. For instance, in discriminant methods [44], Moco V1 [51], and SimCLR [52], we employed the experimental configuration and open-source code in the above literature on all non-implemented labeled data. This paper also trained 1000 epochs on the joint dataset. For simplicity, a KNN classifier was performed on all self-supervised feature learning methods to evaluate the final performance of the classification. It can be seen that the proposed method in this paper was better than other state-of-the-art unsupervised feature learning methods. The proposed method outperformed other unsupervised feature learning methods by about 7.16% in accuracy. It is worth noting that compared with the supervised learning methods trained from scratch, our self-supervised method was pretrained using many unlabeled datasets and fine-tuned only using a small number of labeled datasets. Compared with the supervised learning method trained from scratch, the accuracy of our method achieved 18.85% higher than DenseNet, which had the highest accuracy among all supervised learning methods compared in the experiments. The lowest method was also 5.69% higher than the DenseNet with the highest accuracy in supervised learning, which further proves the effectiveness of the self-supervised learning method in this paper.

### 4.5. Segmentation Results of COVID-19 Lesions

**Figure 4:**
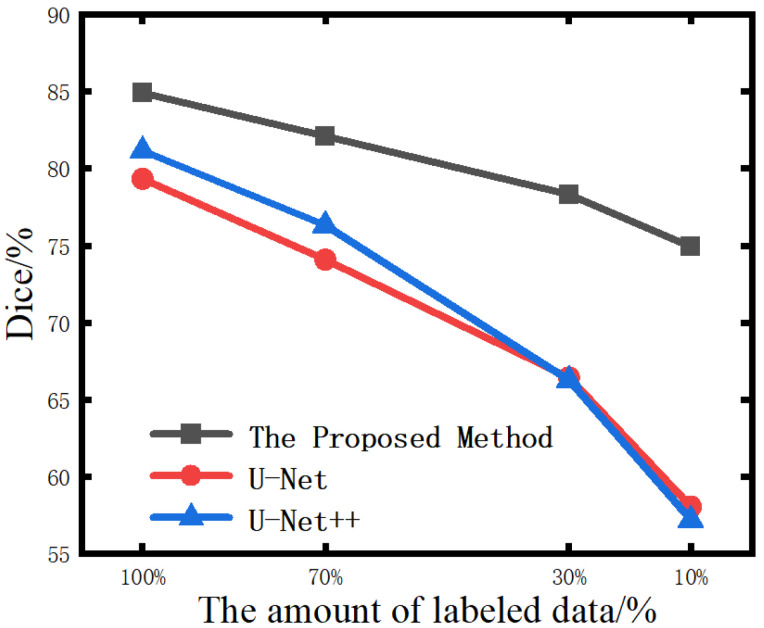
Comparison of lesion segmentation results under different labeled data volumes.

**Figure 5:**
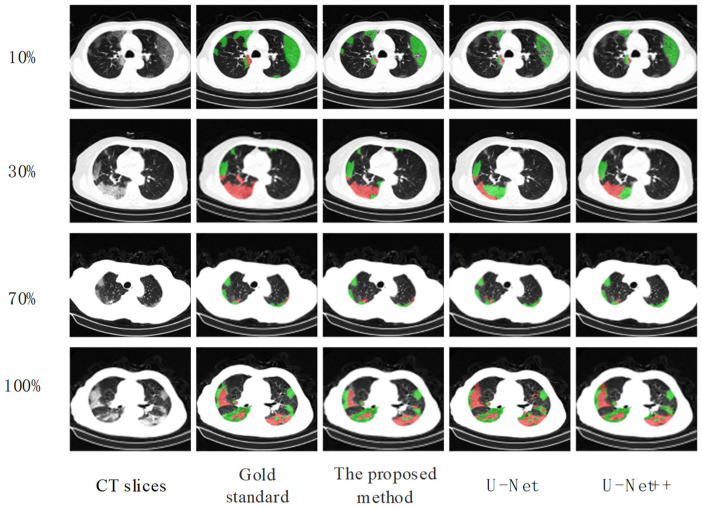
Comparison of Lesion Segmentation Results by Different Methods.

The pre-trained encoder-decoder model is transferred to the downstream lesion segmentation task, and the labeled data set was used for fine-tuning training. The labeled dataset was divided into four proportions, 10%, 30%, 70%, and 100%, respectively. These four sets of data were used to fine-tune the encoder-decoder model to segment the lesion area in the CT images of COVID-19. In addition, each group also experimented in a supervised learning environment. The models under supervised learning adopted the classic segmentation models U-Net and U-Net++ [53] and were trained from scratch using labeled data. The experiment results are shown in Table 4. It can be seen that the greater the amount of data involved in supervised learning training, the smaller the gap between the segmentation accuracy of the proposed method and that of fully supervised learning was. When the amount of data involved in optimizing supervised learning gradually decreased, the performance of the fully supervised learning model gradually declined. The decline rate increased with the reduction of labeled training samples. Although the method used in this paper was only slightly better than other supervised learning in the case of a large amount of data, when the amount of labeled data was reduced to a certain extent, its advantage over supervised learning was getting bigger and bigger. When the amount of labeled data was 100%, as shown in Figure 4, the Dice value of this method was 5.58% higher than that of U-Net.; in 70% of cases, our method is 8.02% higher than U-Net; in 30% of cases, our method is 11.88% higher than U-Net; and in 10% of cases, our method is 16.88%. higher than U-Net. Figure 5 shows the visualization of segmentation results achieved by three different methods on different amounts of annotated data. Under-segmentation and wrong-segmentation results were produced in this case. These results further show the advantages of our method, and the comparative segmentation effects prove that fine-tuning with less data is better than other supervised learning methods.

### 4.6. Ablation Experiment

In this section, extensive ablation studies were conducted to demonstrate the importance of some settings in the model. Several issues were investigated: (1) The effect of data augmentation on the performance of self-supervised learning; (2) The impact of pretext tasks; (3) The impact of the amount of unlabeled data.

#### 4.6.1. Data Augmentation Analysis

We evaluated the importance of augmentation operations by removing them from the transformation set or applying them individually to determine the effect of data augmentation. First, we performed experiments using the model without augmentation. As shown in Table 5, data augmentation had a significant impact on model performance, and a single augmentation operation was not enough to learn discriminative representations. Nonetheless, binarization alone performed much better than other operations, indicating that it enabled the encoder to extract more critical features. We believe the reason lies in that the ground-glass and solid-change gray values in CT images are similar, and more obvious global and local features can be obtained after binarization. It can be seen that the absence of any one of the four augmentation operations led to a decrease in performance and applying a composite data augmentation combination of the four augmentation operations improved the performance of self-supervised learning representations significantly.

#### 4.6.2. Influence of Pretext Tasks

Three pretext tasks were co-trained to analyze the effect of the balance parameters ($\lambda_{1},\lambda_{2},\lambda_{3}$) between the loss functions of each task in the model. When $\lambda_{1}=0,\lambda_{2}=0,\lambda_{3}=0$, the pretext task was not trained. As the values of $\lambda_{1},\lambda_{2},\mathrm{and}\;\lambda_{3}$ increased, the task become more and more important in network training. The experimental settings were the same as those in Section 3.4. When $\lambda_{1}=1,\lambda_{2}=0,$ only the instance discrimination task was trained. When $\lambda_{1}=0,\lambda_{2}=1,$ only rotation prediction task was trained. Due to the irreplaceability of the reconstruction task, this paper set $\lambda_{3}$ to 3. It can be seen from Table 6 that when only the rotation prediction task was trained, we achieved very limited performance, with an accuracy of 78.42%. However, only using instance discrimination task training obtained a better result, with an accuracy of 85.67%. The result indicated that the instance discrimination task, i.e., the contrastive learning method, outperformed other pretext tasks. Through the collaborative training of these two tasks, our method can achieve better classification results. When $\lambda_{1}=1.5,\lambda_{2}=1$, the model achieved the best results with 95.49% accuracy, 93.66% precision, 86.98% recall, and 90.19% F1 score.

#### 4.6.3. The Impact of Unlabeled Sample Data Volume

We pre-trained a self-supervised model using the 3D-COVID private dataset and fine-tuned it on the lesion segmentation datasets (COVID19-seg and CC-COVID) to analyze the impact of the amount of unlabeled data, respectively. We conducted comparative experiments with all datasets and pre-trained using only the unlabeled data of the 3D-COVID dataset. The experimental settings were consistent with Section 4.4 and Section 4.5. As can be seen from Table 7, compared with the models pre-trained with the three unlabeled datasets, the model was pre-trained only with the 3D-COVID unlabeled dataset. The features learned by the model were limited, and the classification and segmentation effects on downstream tasks were general. This also shows that if more unlabeled CT images of pneumonia are available, the performance of the proposed self-supervised method can be further improved, and the generalization ability is also stronger.

## 5. Discussion

COVID-19 was recognized as a pandemic by the World Health Organization on 11 March 2020. The variants it produces are still affecting human health so far. Improving the efficiency of COVID-19 identification is an urgent problem for researchers. Although deep learning has been well developed, the pneumonia image data that can be used for training requires precise human annotation, which means a lot of labor and cost. We may have found a solution to this problem. In this paper, we proposed a self-supervised learning framework, which is used for automatic severity assessment of COVID-19 and lung lesion segmentation in chest CT images. The proposed framework trains the network in a self-supervised manner without relying on a large amount of labeled data. We find and verify that the pretext tasks of decision rotation and multi-instance instance discrimination enable neural networks to learn image features label-free. The results presented in Figure 4 demonstrate the outstanding advantages of our method in the case of limited labeled data. With only 10% annotation, our Dice value is even 17.73% higher than that of U-Net. Perhaps the reason why our methods work is that they help to identify differences in the inherent structure of the lung and the characteristics of the lesions. Rotation prediction helps discover the discriminative structure of CT images by learning rotation-dependent features. Multi-view instance discrimination helps explore rotation-invariant features for COVID-19 lesion segmentation.

Experimental results on an aggregated dataset consisting of three COVID-19 CT image datasets show that our self-supervised method achieved better results than other state-of-the-art methods. We demonstrated a rotation-oriented collaborative pretext task in an AI-based system by using four performance evaluation metrics including (i) severity figure and (ii) dice curve. Figure 5 shows visual binary mask overlays, where red represents the output of the AI model and green represents the foreground (white) region. Due to the pre-training on many unlabeled datasets, there were limited annotations. In the data set scenario, the method in this paper has great advantages compared with supervised learning methods. In the conventional segmentation tasks, researchers focus on a rigorous fit of the model to ensure accurate labeling and prediction outputs, whereas our approach focuses on alleviating the reliance on a large number of markers. Compared with enhancing the model effect by pre-training with general classification datasets, the features learned by our method are more suitable for medical scenarios because our training data are all from medical images. Although our method achieves impressive results, it still suffers from some limitations. The features learned by the model are independent in 3D, and the connections between each lung slice are not counted. This may reduce the performance of the algorithm. Furthermore, it still relies on marking some very precise annotations, which still has a non-negligible amount of work. We also possibly combine learning-based algorithm with traditional segmentation algorithms to improve the performance [54,55]. Anyway, the development of artificial intelligence (AI)-based solutions specific to COVID-19 identification and severity quantification could provide a fast, efficient, and reliable alternative. This approach complements traditional medical diagnostic strategies and accelerates research in image analysis while reducing the burden on physicians.

## 6. Conclusions

This article proposes a self-supervised learning method for lesion segmentation and disease severity grading for COVID-19. The main idea of this algorithm is to learn visual features from many unlabeled images by developing joint rotation-oriented tasks, namely rotation prediction tasks and multi-view instance discrimination tasks. The rotation prediction task helps to discover the discriminative structure of COVID-19 CT images by learning rotation-related features, while the multi-view instance discrimination task helps to explore rotation-invariant features in COVID-19 images. These two features, namely rotation-dependent features and rotation-invariant features are obtained by co-training two pre-tasks. Experimental results on three datasets show that our method outperforms state-of-the-art SSL methods. Due to the availability of a large amount of unlabeled data, our method can exceed the baseline of pure segmentation tasks and is very close to the baseline of severity classification, showing that our method can be used in clinical potential benefits.

The future research direction of this paper is to design a more efficient excuse learning method and make full use of unlabeled data to solve the problem of scarcity of labeled medical image datasets.

## Figures and Tables

**Table 1:** Encoder-Decoder Network Architecture.

Module Name	Num	Floor	Input	The Amount of Data
Encoder block 1	2	{conv, batchnorm, ReLU}	2D CT slices	34 K
pooling layer 1	1	max-pooling	Encoder block 1	-
Encoder block 2	2	{conv, batchnorm, ReLU}	pooling layer 1	68 K
pooling layer 2	1	max-pooling	Encoder block 2	-
Encoder block 3	2	{conv, batchnorm, ReLU}	pooling layer 2	68 K
pooling layer 3	1	max-pooling	Encoder block 3	-
Encoder block 4	2	{conv, batchnorm, ReLU}	pooling layer 3	68 K
pooling layer 4	1	max-pooling	Encoder block 4	-
Encoder block 5	2	{conv, batchnorm, ReLU}	pooling layer 4	2465 K
Decoder-block 4	1	{up-sample, conv, batchnorm, ReLU, concat}	Encoder block 5	358 K
	2	{conv, batchnorm, ReLU}	Decoder-block 4	
Decoder-block 3	1	{up-sample, conv, batchnorm, ReLU, concat}	Decoder-block 4	137 K
	2	{conv, batchnorm, ReLU}	Decoder-block 3	
Decoder-block 2	1	{up-sample, conv, batchnorm, ReLU, concat}	Decoder-block 3	137 K
	2	{conv, batchnorm, ReLU}	Decoder-block 2	
Decoder-block 1	1	{up-sample, conv, batchnorm, ReLU, concat}	Decoder-block 2	137 K
	1	{conv, batchnorm, ReLU}	Decoder-block 1	
1 × 1 Conv block	1	1 × 1 conv	Decoder-block 1	0.25 K

**Table 2:** Annotate the number of samples of different severity in the dataset.

Datasets	3D-COVID	COVID19-Seg	CC-COVID
Slight	200	60	47
Medium	250	30	29
Severe	150	10	24
Sum	600	100	100

**Table 3:** Comparison of Different Methods in COVID-19 Severity Classification Experiment.

	Method	Accuracy	Precision	Recall	F1 Score
Supervised	VGG19 [48]	73.21	68.60	63.26	65.82
	ResNet50 [36]	75.89	73.19	69.58	71.34
	DenseNet121 [49]	76.64	78.51	72.95	75.62
Self-supervised	Rotation [50]	82.33	78.39	65.00	71.06
	Wu [44]	84.69	88.63	74.34	80.85
	Moco V1 [51]	88.33	82.49	70.53	76.04
	SimCLR [52]	84.21	79.17	71.88	75.34
	Ours	**95.49**	**93.66**	**86.98**	**90.19**

**Table 4:** Comparison of Dice coefficients of three segmentation methods under different labeled data volumes.

Labels	Method	Dice %
10%	Ours	**74.94**
	U-Net	58.06
	U-Net++	57.21
30%	Ours	**78.32**
	U-Net	66.44
	U-Net++	66.27
70%	Ours	**82.11**
	U-Net	74.09
	U-Net++	76.32
100%	Ours	**84.91**
	U-Net	79.33
	U-Net++	81.16

**Table 5:** Comparison of Dice Coefficients for Different Enhanced Combinations.

Crop	Color Jitter	Random Erasing	Binarization	Dice
				77.12
✓				79.44
	✓			78.79
		✓		77.89
			✓	**79.22**
✓	✓	✓		80.83
✓		✓	✓	81.66
✓	✓		✓	81.19
	✓	✓	✓	82.91
✓	✓	✓	✓	**84.91**

**Table 6:** Comparison of models under different parameters.

	Accuracy	Precision	Dice	F1 Score
*λ*_1_ = 1, *λ*_2_ = 0	85.67	83.95	76.51	80.05
*λ*_1_ = 1, *λ*_2_ = 0.5	87.69	84.42	80.98	82.66
*λ*_1_ = *λ*_2_ = 1	89.03	87.12	82.37	84.67
*λ*_1_ = 1.5, *λ*_2_ = 1	**95.49**	**93.66**	**86.98**	**90.19**
*λ*_1_ = 2, *λ*_2_ = 1	92.21	88.55	81.23	84.73
*λ*_1_ = 0, *λ*_2_ = 1	78.42	76.97	69.38	72.98

**Table 7:** Comparison of pre-training experiments with different data volumes.

Pre-Training	Accuracy	Precision	Recall	F1 Score	Dice
All	95.49	93.66	86.98	90.19	84.91
Dataset 1	83.52	79.19	78.30	78.74	75.26
